# Supplementary material for: An improved d-band model of the catalytic activity of magnetic transition metal surfaces
Source: Sci Rep. 2016 Nov 3;6:35916. doi: 10.1038/srep35916 (PMC5093898; doi:10.1038/srep35916)
Supplement: Supplementary Information [file srep35916-s1.pdf]

Supplemental material of
"An improved d-band model of the catalytic activity of magnetic transition metal surfaces"

S. Bhattacharjee[1], U. V. Waghmare[2] and S. C. Lee[1,3]

[1]*Indo-Korea Science and Technology Center (IKST), Bangalore, India*

[2]*Jawaharlal Nehru Centre for Advanced Scientific Research(JNCASR), Bangalore, India*

[3]*Electronic Materials Research Center,*

Korea Institute of Science & Tech, Korea

Derivation of two-centered d band model

Let us consider the simplest case, an adsorbate with a single molecular orbital is interacting with a transition metal (TM) surface represented by its d-band centers $\varepsilon_{d\sigma}$. σ is the spin index($\sigma = \uparrow, \downarrow$). Supposing that the metal and the adsorbate sub-systems are characterized by the wave functions respectively $\Psi_{d\sigma}$ and $\Psi_{a\sigma}$, i,e

$$\mathcal{H}_d \Psi_{d\sigma} = \varepsilon_{d\sigma} \Psi_{d\sigma} \tag{1}$$

and

$$\mathcal{H}_a \Psi_{a\sigma} = \varepsilon_{a\sigma} \Psi_{a\sigma} \tag{2}$$

when they are not coupled. $\varepsilon_{a\sigma}$ is the energy of the adsorbate state with spin σ. The wavefunction of the interacting system , $\mathcal{H}_{tot} = \mathcal{H}_d + \mathcal{H}_a + \mathcal{H}_{da}$, (where $\mathcal{H}_{da}|\Psi_{d\sigma} >= V|\Psi_{a\sigma} >$ describes the mixing between a and d states) can be written in terms of linear combination of atomic orbitals (LCAO) approach,

$$\Psi_{da\sigma} = C_{d\sigma}\Psi_{d\sigma} + C_{a\sigma}\Psi_{a\sigma} \tag{3}$$

the energy of the interacting system is given by,

$$E = \frac{< \Psi_{da\sigma}|\mathcal{H}_{tot}|\Psi_{da\sigma} >}{< \Psi_{da\sigma}|\Psi_{da\sigma} >} \tag{4}$$

Due to the interaction there will be spin-dependent bonding and anti-bonding orbitals which can be obtained by setting:

$$\frac{\partial E}{\partial C_{d\sigma}} = 0$$

and

$$\frac{\partial E}{\partial C_{a\sigma}} = 0$$

$$\begin{aligned}
\varepsilon_{b\uparrow} &= \frac{\varepsilon_{d\uparrow}+\varepsilon_a}{2} - VS - \sqrt{\frac{4V^2+(\varepsilon_{d\uparrow}-\varepsilon_a)^2}{2}}, \\
\varepsilon_{ab\uparrow} &= \frac{\varepsilon_{d\uparrow}+\varepsilon_a}{2} - VS + \sqrt{\frac{4V^2+(\varepsilon_{d\uparrow}-\varepsilon_a)^2}{2}}, \\
\varepsilon_{b\downarrow} &= \frac{\varepsilon_{d\downarrow}+\varepsilon_a}{2} - VS - \sqrt{\frac{4V^2+(\varepsilon_{d\downarrow}-\varepsilon_a)^2}{2}}, \\
\varepsilon_{ab\downarrow} &= \frac{\varepsilon_{d\downarrow}+\varepsilon_a}{2} - VS + \sqrt{\frac{4V^2+(\varepsilon_{d\downarrow}-\varepsilon_a)^2}{2}}
\end{aligned} \tag{5}$$

Where we have considered $\varepsilon_{a\uparrow} = \varepsilon_{a\downarrow} = \varepsilon_a$. $V =< \Psi_{d\sigma}|\mathcal{H}_{da}|\Psi_{a\sigma} >$, are independent of the spin. The subscript 'b' refers the bonding state, while 'ab' refers the anti-bonding state. The situation is shown in the Fig.1 of the manuscript. The overlap integral is defined by $S =< \Psi_{d\sigma}|\Psi_{a\sigma} >$, again same for the both spin components for a given TM.

Case-1,the adsorbate orbital is occupied, $\varepsilon_a < \varepsilon_{d\sigma}$

Suppose that the metal state is having the fractional occupancies respectively for the two $f_\uparrow$ and $f_\downarrow$ for the two spin channels, In this case, the change in energy due to the adsorbate-metal interaction can be written as,

$$\begin{aligned}
\Delta E_d =& (\varepsilon_{b\uparrow} + \varepsilon_{b\downarrow} + f_\uparrow \varepsilon_{ab\uparrow} + f_\downarrow \varepsilon_{ab\downarrow}) - 2\varepsilon_a - f_\uparrow \varepsilon_{d\uparrow} - f_\downarrow \varepsilon_{d\downarrow} \\
&= -(1-f_\uparrow)\frac{V^2}{\varepsilon_{d\uparrow}-\varepsilon_a} - (1-f_\downarrow)\frac{V^2}{\varepsilon_{d\downarrow}-\varepsilon_a} + (1+f_\uparrow)\alpha V^2 + (1+f_\downarrow)\alpha V^2
\end{aligned} \tag{6}$$

Where $\alpha = -\frac{S}{V}$.

Case-2,the adsorbate orbital is unoccupied, $\varepsilon_a > \varepsilon_{d\sigma}$

The change in energy in this case is,

$$\Delta E_d = -f_\uparrow \frac{V^2}{\varepsilon_a - \varepsilon_{d\uparrow}} - f_\downarrow \frac{V^2}{\varepsilon_a - \varepsilon_{d\downarrow}} + f_\uparrow \alpha V^2 + f_\downarrow \alpha V^2 \tag{7}$$

In the case of NH_3 molecule, we have 4 adsorbate orbitals, two among them are occupied(HOMO) and the remaining two are empty(LUMO), leading us to

$$\Delta E_d = \underbrace{-\sum_{\sigma,i} f_\sigma \frac{V^2}{\varepsilon_i - \varepsilon_{d\sigma}}}_{\text{d-LUMO interaction}} \underbrace{-\sum_{\sigma,j}(1-f_\sigma)\frac{V^2}{\varepsilon_{d\sigma} - \varepsilon_j}}_{\text{d-HOMO interaction}} \underbrace{+\sum_{\sigma,i} f_\sigma \alpha V^2}_{\text{d-LUMO orthogonalization}} \underbrace{+\sum_{\sigma,j}(1+f_\sigma)\alpha V^2}_{\text{d-HOMO orthogonalization}} \tag{8}$$

Here the subscripts 'i' and 'j' respectively refers to LUMO and HOMO regions of ε_a.

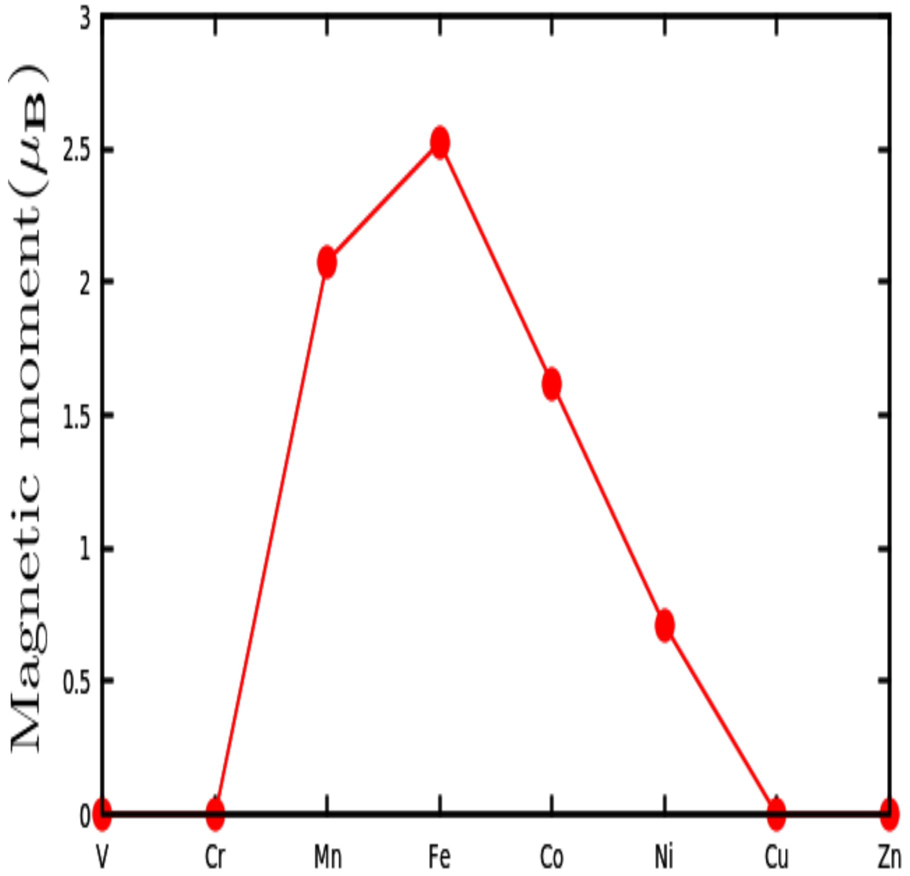

FIG. 1: (Color online) The magnetic moment of the TM atom attached to NH_3 for different TM-surfaces

Origin of attractive and repulsive interactions: simple picture

To understand the interaction described by Eq.(2) of the manuscript, we consider a simplified picture with a filled adsorbate state interacting with valence states of the TM-metal. Since there is no empty adsorbate state, the first and third term in the equation (2) are zero and we can write, $\Delta E_d = -\sum_{\sigma,\sigma'}(1-f_\sigma)\frac{{V^{da}_{\sigma,\sigma'}}^2}{|\varepsilon_{d\sigma}-\varepsilon_{a\sigma'}|} + \sum_{\sigma,\sigma'}\alpha(1+f_\sigma){V^{da}_{\sigma,\sigma'}}^2 = (A_\uparrow + A_\downarrow) + (R_\uparrow + R_\downarrow)$. Where A_σ and R_σ are the spin dependent attractive and repulsive part of the metal-adsorbate interaction respectively.

$$\Delta E_d = (A_\uparrow + A_\downarrow) + (R_\uparrow + R_\downarrow) \qquad (9)$$

where

$$A_\sigma = -\sum_{\sigma'} \frac{{V^{da}_{\sigma,\sigma'}}^2}{|\varepsilon_{d\sigma} - \varepsilon_{a\sigma'}|}$$

is the energy gain due to formation of bonding orbitals for spin σ. Similarly,

$$R_\sigma = \sum_{\sigma'} f_\sigma \frac{{V^{da}_{\sigma,\sigma'}}^2}{|\varepsilon_{d\sigma} - \varepsilon_{a\sigma'}|} + \sum_{\sigma'} \alpha(1+f_\sigma){V^{da}_{\sigma,\sigma'}}^2$$

is the repulsive energy due to the formation of antibonding orbitals for spin σ *plus* the energy due to the orthogonalization of adsorbate state to the metal. Let us now consider an hypothetical case: the same TM can exist in two spin polarized states: (I) It is 100% spin polarized, i,e spin-$\uparrow$ states are completely filled while spin-$\downarrow$ states are completely empty. Let us consider the interaction of the filled adsorbate state with such states:

Case I, half-metallic (HM) limit:

Let us consider that the TM is 100% spin polarized i,e the majority spin states are completely filled and the minority spin states are completely empty.

Such situation is represented in the Fig.2a (top panel). In such case, the entire majority spin channel is pushed down so much that there is practically no attractive contribution from the majority spin ($A_{\uparrow} = 0$). Similarly, there is no repulsive contribution from the minority spin ($R_{\downarrow} = 0$), since we have assumed that the down-spin states are completely empty. From Eq. (9) we get,

$$\Delta E_d^{HM} = R_{\uparrow} + A_{\downarrow}, \tag{10}$$

where ΔE_d^{HM} is the adsorption energy for the half-metallic case.

Case II, non-magnetic limit:

In this case, both spin channels contribute equally to the attractive and repulsive part (such situation in this case is represented in the Fig.2b (bottom panel)), and therefore , $A_{\uparrow} = A_{\downarrow}$ and $R_{\uparrow} = R_{\downarrow}$, From Eq. (10), we get

$$\Delta E_d^{NSP} = 2(R_{\uparrow} + A_{\downarrow}) = 2\Delta E_d^{HM}, \tag{11}$$

The adsorption energy for the non-magnetic case, ΔE_d^{NSP} is thus twice in magnitude compared to the adsorption energy in the half-metallic case. It is clear why a *two-band centered d-band model* with appropriate filling is required to predict the catalytic activity on magnetically active surface. Calculation based on single centred model for spin polarized surfaces may lead over-binding(up to a factor of 2).

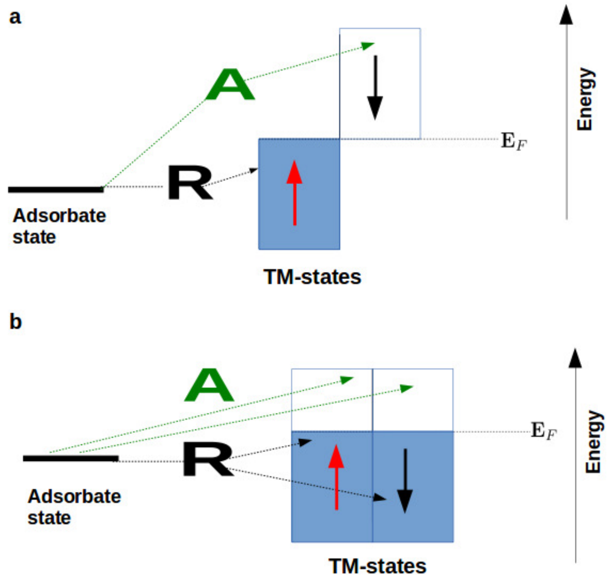

FIG. 2: (Color online) Interaction of a filled adsorbate state with the TM-states in two extream cases: (a)100% spin polarization (half-metallic limit) (b) 0% spin polarization (non-magnetic limit). Here "A" represents attractive interaction while "R" represent repulsive interaction between adsorbate-TM interactions.
